# Editorial: Development of Executive Function during Childhood

**DOI:** 10.3389/fpsyg.2016.00006

**Published:** 2016-01-20

**Authors:** Yusuke Moriguchi, Nicolas Chevalier, Philip D. Zelazo

**Affiliations:** ^1^Department of School Education, Joetsu University of EducationJoetsu, Japan; ^2^Department of Psychology, The University of EdinburghEdinburgh, UK; ^3^Institute of Child Development, University of MinnesotaMinneapolis, MN, USA

**Keywords:** executive function, cognitive flexibility, inhibitory control, working memory, brain development, socio-emotional development, prefrontal cortex (PFC), cognitive development

Executive function (EF) is one of the most rapidly expanding research fields in the developmental and cognitive sciences. The aim of this Research Topic is to present a broad sample of recent advances in understanding the development of EF. The 38 articles in this collection provide a unique, state-of-the-art tour of current, burning issues regarding executive function development, from cutting-edge research on the underpinning basic cognitive processes to the most promising applications in educational and clinical settings.

## Cognitive processes of EF during childhood

EF involves several complex cognitive processes, including working memory, inhibitory control, and cognitive flexibility. The present papers shed new light on how these processes develop and how they are interrelated. Specifically, they clarify the conditions that modulate EF demands (FitzGibbon et al.; Unger et al.), how their effect can persist in time (Garcia and Dick), the specific executive processes (e.g., inhibitory control) at play in a given task (Wright and Diamond) and the specific age windows during which critical changes in EF engagement occur (Lucenet and Blaye). Furthermore, they provide new evidence that EF may develop through progressive differentiation of executive processes from more basic cognitive processes (e.g., processing speed and short-term memory; Clark et al.; Visu-Petra et al.) and of different forms of EF (e.g., cognitive “cool” EF vs. affective “hot” EF) (Gandolfi et al.; Mulder et al.). They further identify the brain correlates of EF development using EEG/ERP or MRI (Checa et al.; Harms et al.; Unger et al.), revealing for instance that anatomical coupling between the left prefrontal cortex and other distributed brain regions predicts behavioral performance (Lee et al.).

## The critical role of EF in social, emotional, and cognitive development

The present papers also reveal or clarify the association of EF to a host of social and emotional processes including, for instance, theory of mind (Austin et al.), referent assignment (Murakami and Hashiya), conversational pragmatics (Blain-Brière et al.), narrative skills (Friend and Bates), prosocial behaviors (Güroǧlu et al.), social interactions (Moriguchi), sensation seeking (Harms et al.), emotional experience (Ferrier et al.), fear (Susa et al.), and emotional overeating (Groppe and Elsner). Impressively, these associations are often found over and above associations with IQ. Other findings highlight links between EF and motor function (Gonzalez et al.), source monitoring (Earhart and Roberts), and conceptual development (Houdé and Borst). These impressive findings highlight the foundational role that EF plays in goal-directed behavior across a wide range of domains and situations, and they underscore that the healthy development of EF skills is critical for both social-emotional and cognitive development. Indeed, they suggest that understanding the development of EF is absolutely key to understanding child development overall.

## EF and academic achievement

One of the most important foci in research on EF is the relation of EF development to school readiness and academic achievement. The studies included in this Research Topic provide further evidence of the predictive value of EF in academic learning, and in particular reading (Engel de Abreu et al.). Critically, they also clarify the discriminative importance of EF processes for children's mathematical learning, showing how the role of EF may increase from preschool to kindergarten (Clark et al.; McClelland et al.) and then wane in adolescence (Boschloo et al.). Such findings charting out the influence of EF on academic learning are essential to designing effective interventions that target strategic time points in development. Indeed, extant evidence suggests that such training programs can effectively enhance academic achievement (Karbach and Unger; Segretin et al.), although socio-environmental factors, such as housing conditions, may moderate the effects of cognitive interventions in children (Segretin et al.).

## Experiences affecting EF

Given the importance of EF for child development and academic achievement, several studies examined experiential influences that may affect its development. The findings suggest that some activities, such as regular energy drink consumption during adolescence, may *impair* EF (Batenburg-Eddes et al.), whereas others, such as time spent in non-structured activities, may *promote* it during childhood (Barker et al.). Meanwhile, the influence of other factors that have long been assumed to affect EF, in particular bilingualism, may have been overestimated in the past (Gathercole et al.). All these thought-provoking findings have important implications on societal choices and for policy makers.

## EF disorders

Just as EF appears to play an essential role in typical development, difficulties in EF are central features of several developmental disorders. The studies in this Research Topic contribute to clarifying the role of EF in ADHD symptoms (Lahat et al.), Chromosome 22q11.2 Deletion Syndrome (Shapiro et al.), and severe speech and motor impairments (Stadskleiv et al.).

## Measuring EF in children

Finally, advances in research on EF development rely critically on designing effective, valid, and reliable instruments and methodologies. The present papers greatly contribute to this effort by developing new EF tasks (Ikeda et al.) and showing how physiological measures, such as pupil dilation and phasic heart rate variability (HRV), can bring further insight on children's EF (Byrd et al.; Johnson et al.).

## Summary

The articles in this Research Topic demonstrate how considerations of both basic cognitive/biological processes and applied/clinical settings help to unify and extend our understanding of EF during childhood. They illustrate the large range of questions and debates that animate this particularly dynamic field. We hope that this Research Topic will be helpful to both novices and experts of EF development by providing an overview of the field and highlighting the most recent advances.

## Author contributions

All authors drafted the manuscript, and provided critical revisions. All authors approved the final version of the manuscript for submission.

### Conflict of interest statement

The authors declare that the research was conducted in the absence of any commercial or financial relationships that could be construed as a potential conflict of interest.

